# Supplementary material for: Machine Learning Analysis Identifies Drosophila Grunge/Atrophin as an Important Learning and Memory Gene Required for Memory Retention and Social Learning
Source: G3 (Bethesda). 2017 Sep 9;7(11):3705–18. doi: 10.1534/g3.117.300172 (PMC5677163; doi:10.1534/g3.117.300172)
Supplement: Supplementary file 1 [file 3705FigureS1.pptx]

## Slide 1
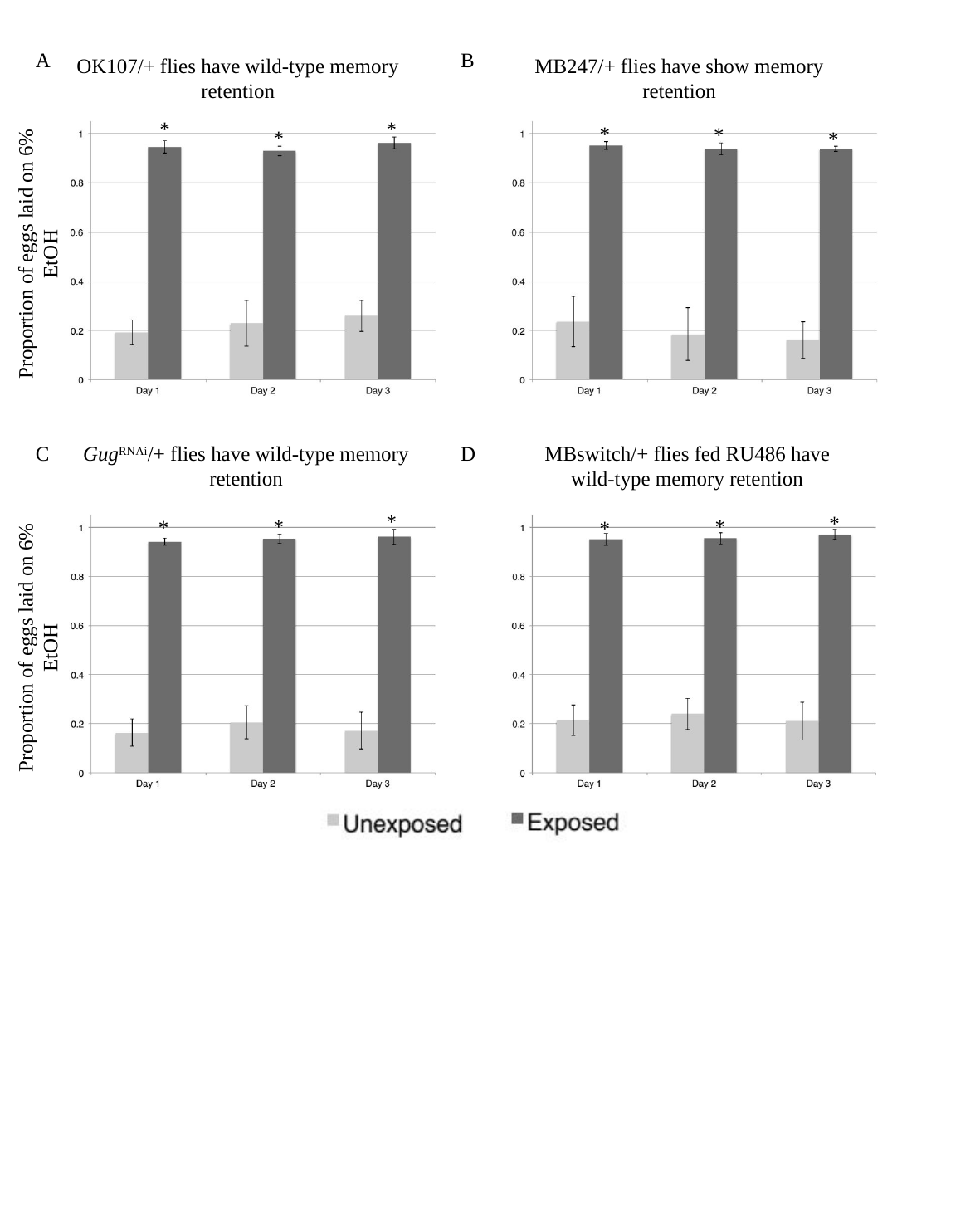

A
B
OK107/+ flies have wild-type memory retention
MB247/+ flies have show memory retention
*
*
*
*
*
*
Proportion of eggs laid on 6% EtOH
C
GugRNAi/+ flies have wild-type memory retention
D
MBswitch/+ flies fed RU486 have wild-type memory retention
*
*
*
*
*
*
Proportion of eggs laid on 6% EtOH
