# Supplementary figures and images for: Machine Learning Analysis Identifies Drosophila Grunge/Atrophin as an Important Learning and Memory Gene Required for Memory Retention and Social Learning
Source: G3 (Bethesda). 2017 Sep 9;7(11):3705–18. doi: 10.1534/g3.117.300172 (PMC5677163; doi:10.1534/g3.117.300172)

## Slide 1
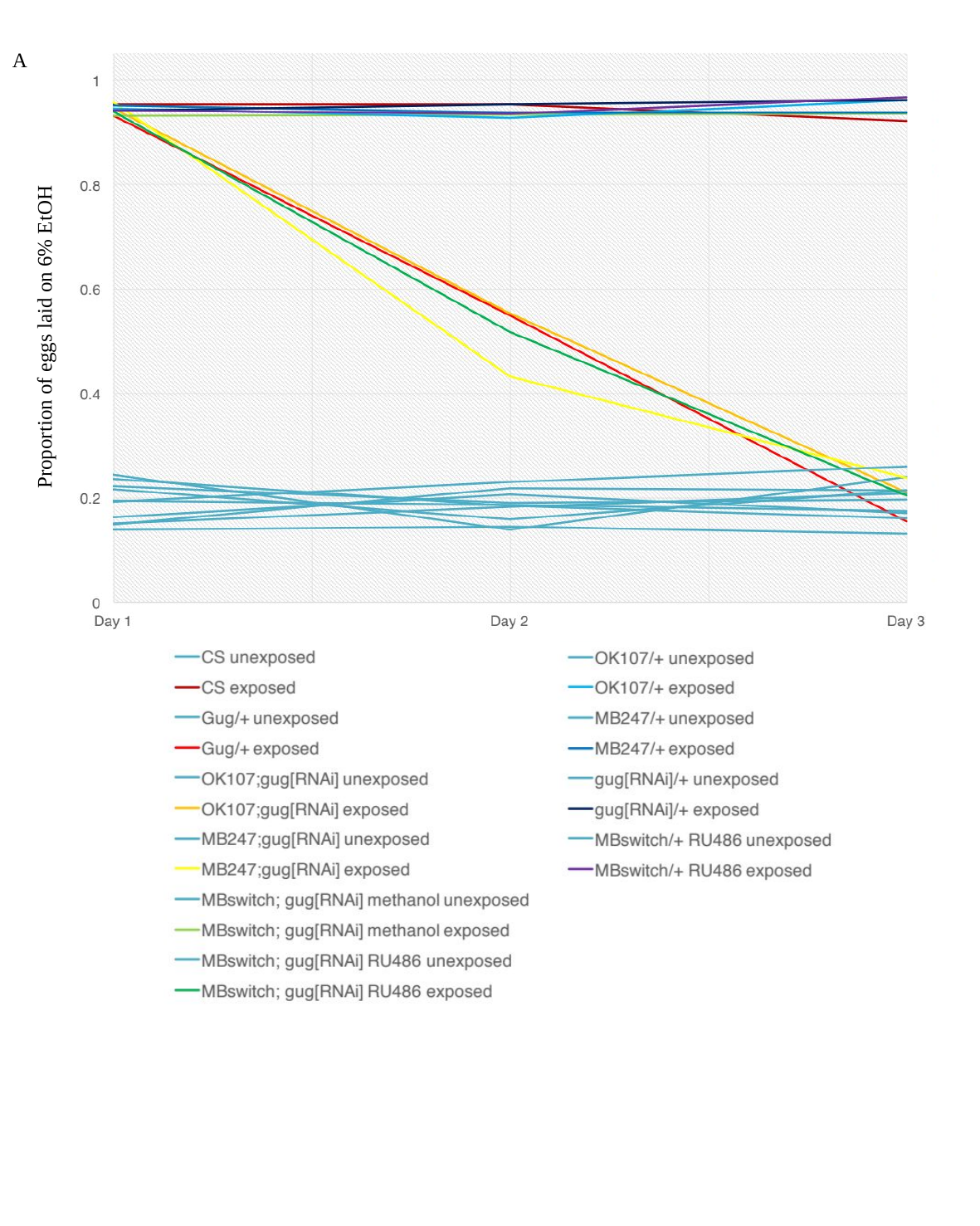

A
Proportion of eggs laid on 6% EtOH

Supplement: Supplementary file 2 [file 3705FigureS2.pptx]

## Slide 1
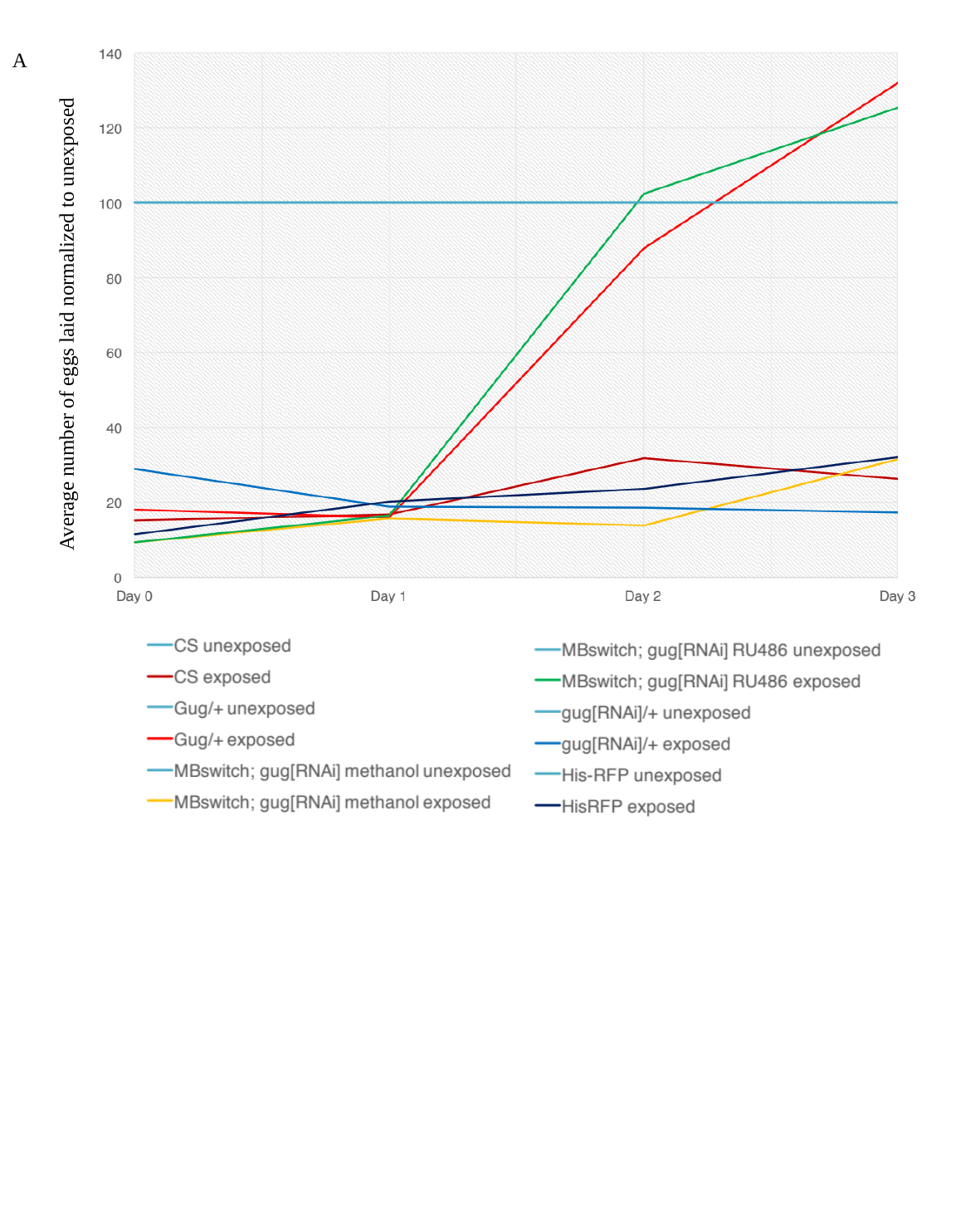

A
Average number of eggs laid normalized to unexposed

Supplement: Supplementary file 4 [file 3705FigureS4.pptx]

## Slide 1
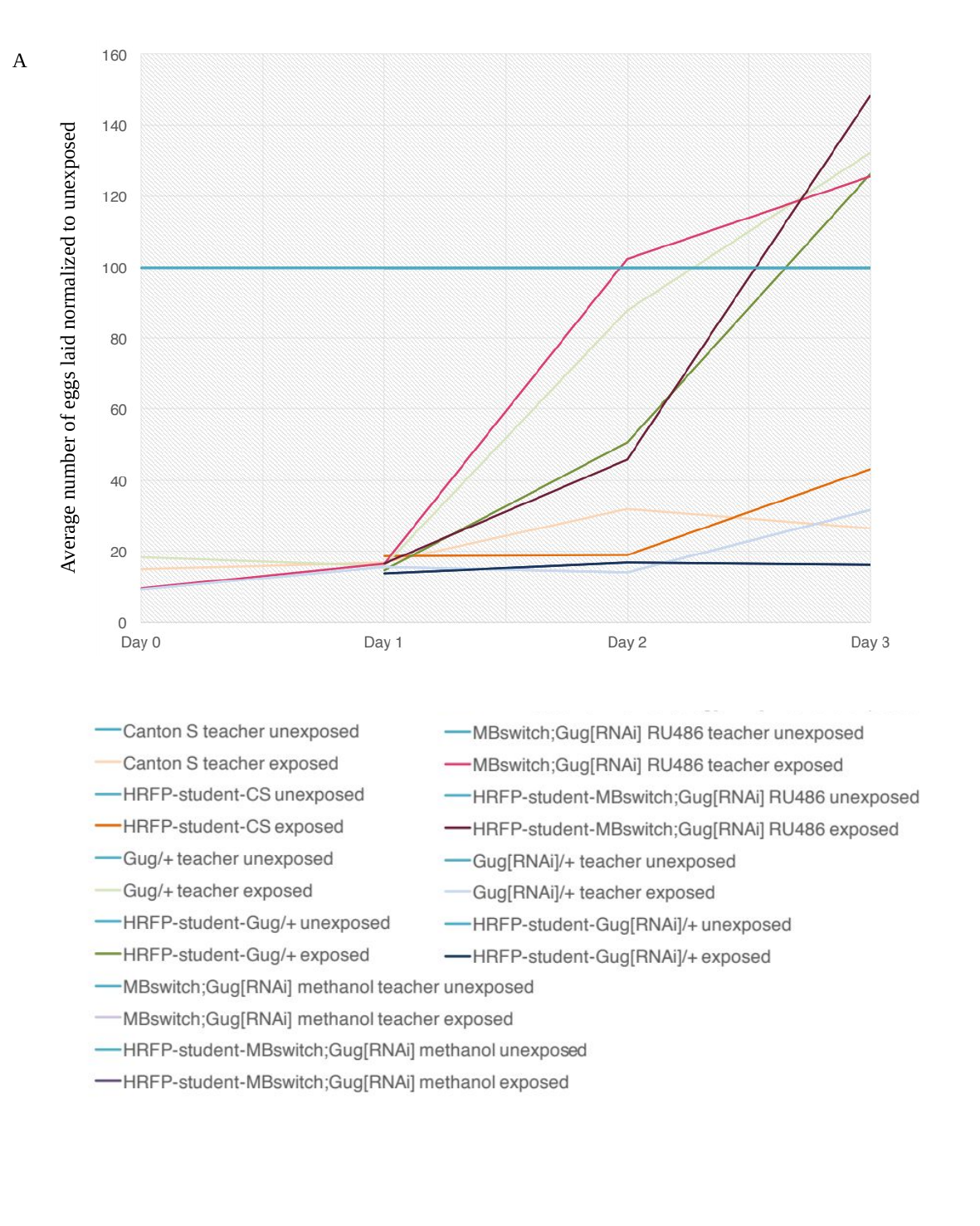

A
Average number of eggs laid normalized to unexposed

Supplement: Supplementary file 6 [file 3705FigureS6.pptx]

## Slide 1
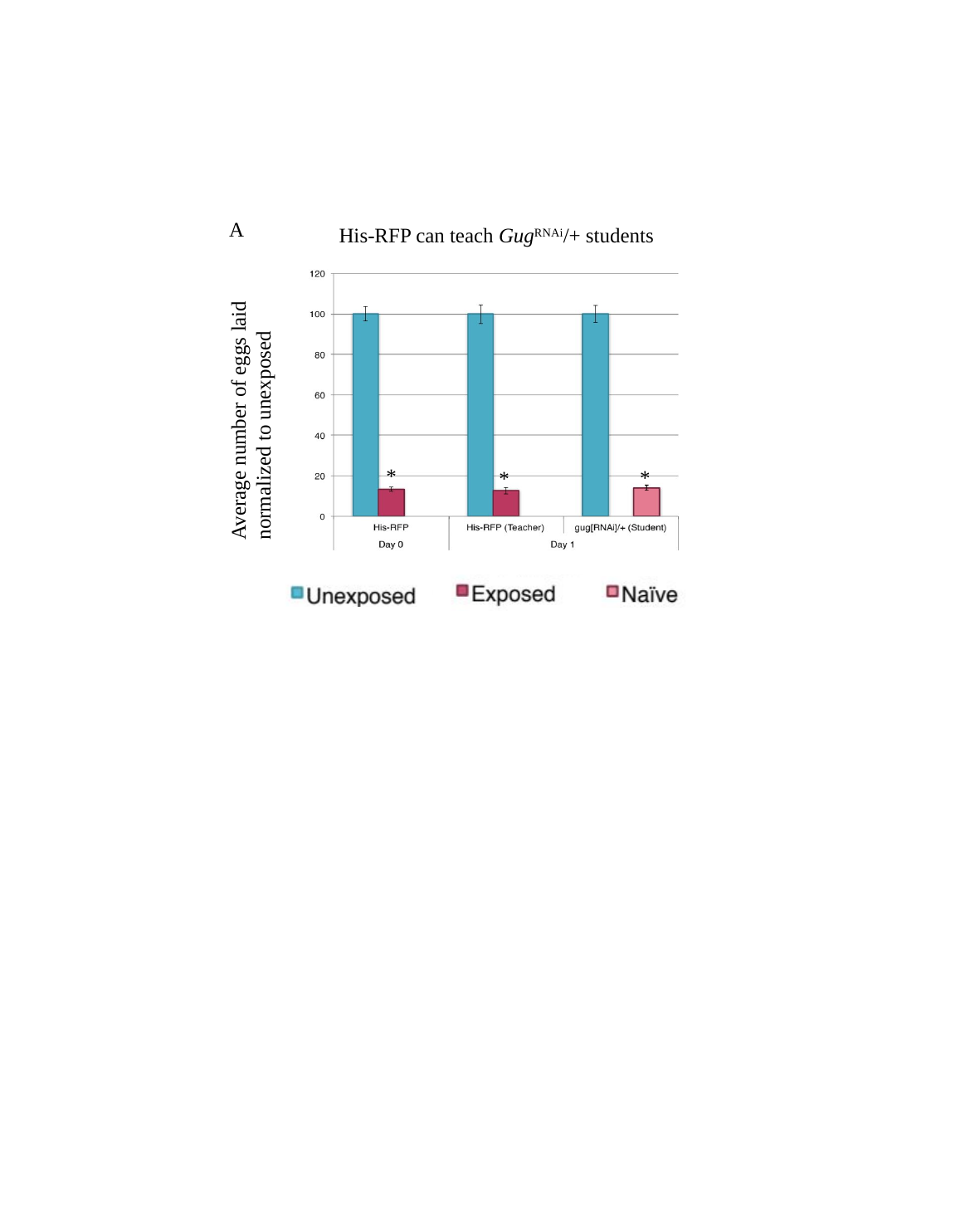

A
His-RFP can teach GugRNAi/+ students
Average number of eggs laid normalized to unexposed
*
*
*

Supplement: Supplementary file 7 [file 3705FigureS7.pptx]

## Slide 1
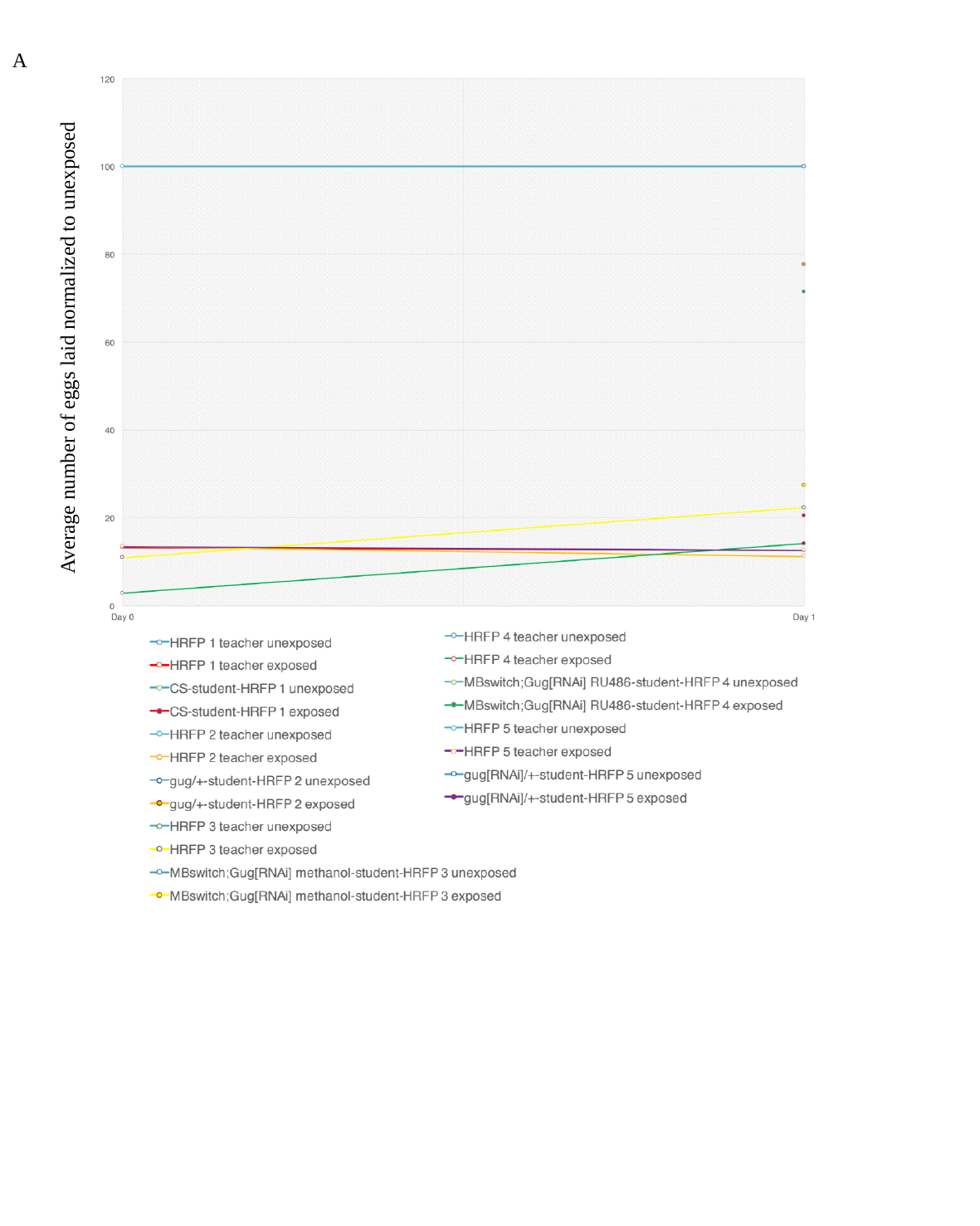

A
Average number of eggs laid normalized to unexposed

Supplement: Supplementary file 8 [file 3705FigureS8.pptx]
