# Supplementary material for: Machine Learning Analysis Identifies Drosophila Grunge/Atrophin as an Important Learning and Memory Gene Required for Memory Retention and Social Learning
Source: G3 (Bethesda). 2017 Sep 9;7(11):3705–18. doi: 10.1534/g3.117.300172 (PMC5677163; doi:10.1534/g3.117.300172)
Supplement: Supplementary file 3 [file 3705FigureS3.pptx]

## Slide 1
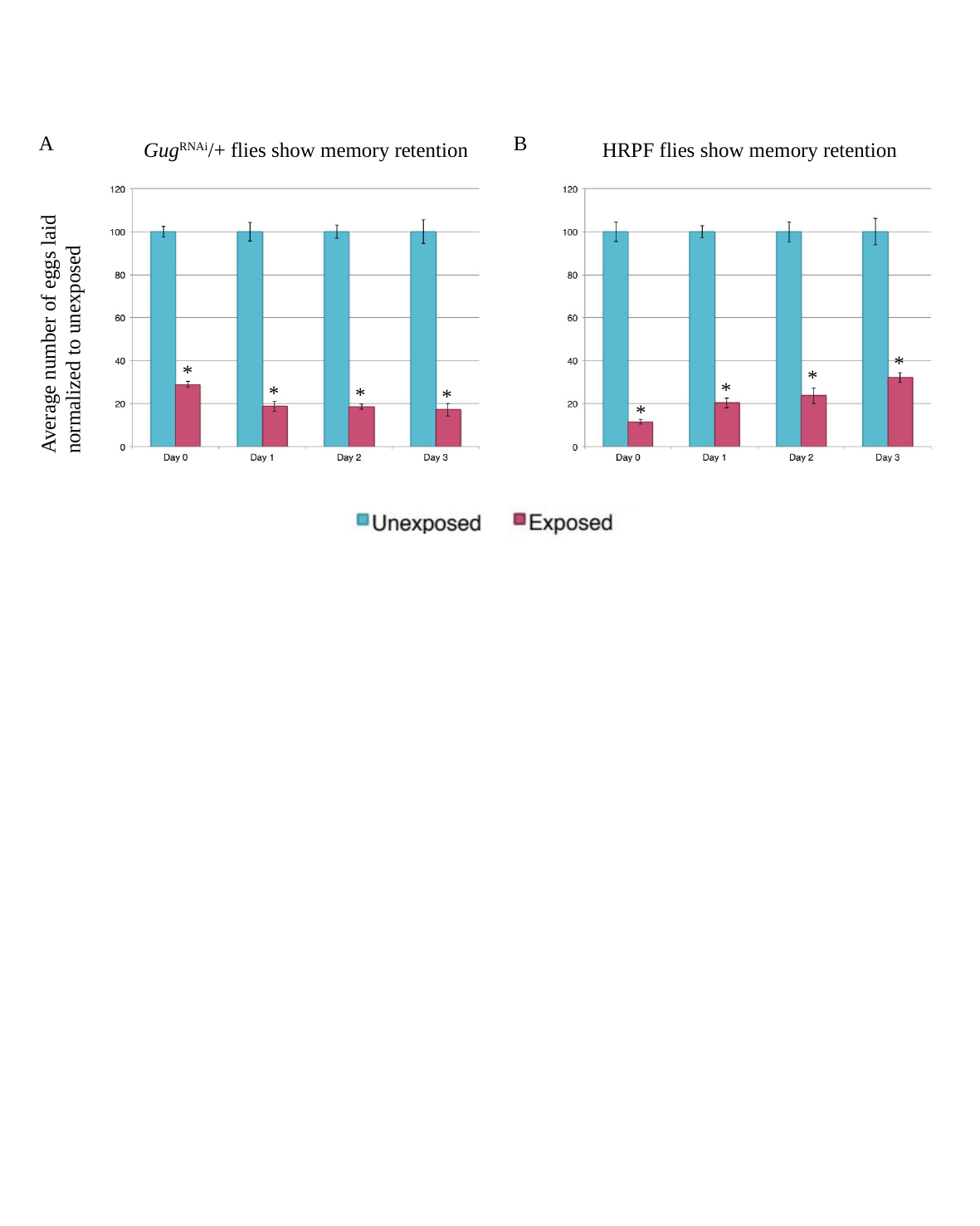

A
B
GugRNAi/+ flies show memory retention
HRPF flies show memory retention
Average number of eggs laid normalized to unexposed
*
*
*
*
*
*
*
*
