# Supplementary material for: Machine Learning Analysis Identifies Drosophila Grunge/Atrophin as an Important Learning and Memory Gene Required for Memory Retention and Social Learning
Source: G3 (Bethesda). 2017 Sep 9;7(11):3705–18. doi: 10.1534/g3.117.300172 (PMC5677163; doi:10.1534/g3.117.300172)
Supplement: Supplementary file 9 [file 3705TableS1.pptx]

## Slide 1
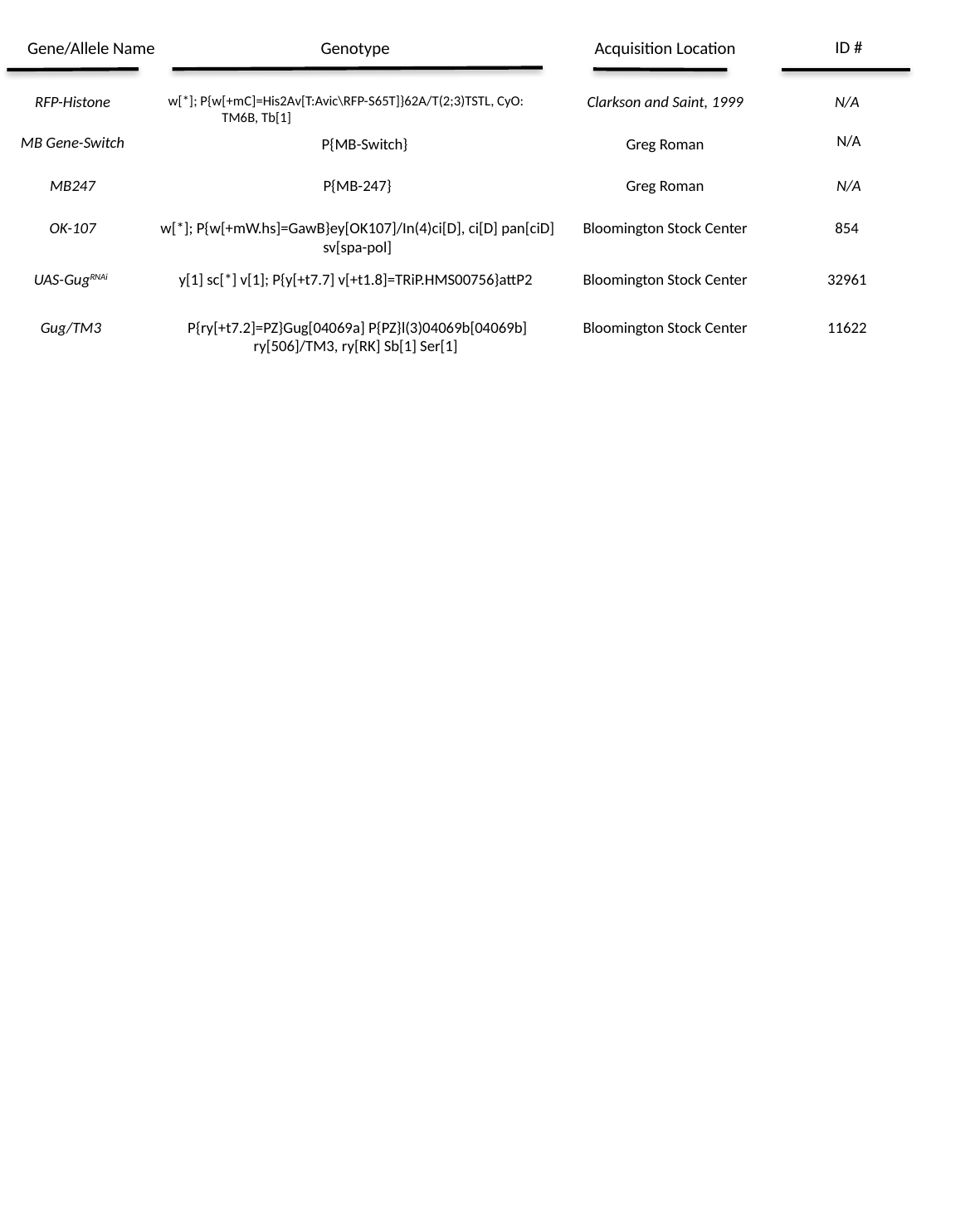

ID #
Genotype
Acquisition Location
Gene/Allele Name
RFP-Histone
w[*]; P{w[+mC]=His2Av[T:Avic\RFP-S65T]}62A/T(2;3)TSTL, CyO: TM6B, Tb[1]
Clarkson and Saint, 1999
N/A
N/A
MB Gene-Switch
Greg Roman
P{MB-Switch}
MB247
P{MB-247}
Greg Roman
N/A
OK-107
w[*]; P{w[+mW.hs]=GawB}ey[OK107]/In(4)ci[D], ci[D] pan[ciD] sv[spa-pol]
Bloomington Stock Center
854
UAS-GugRNAi
y[1] sc[*] v[1]; P{y[+t7.7] v[+t1.8]=TRiP.HMS00756}attP2
Bloomington Stock Center
32961
Gug/TM3
P{ry[+t7.2]=PZ}Gug[04069a] P{PZ}l(3)04069b[04069b] ry[506]/TM3, ry[RK] Sb[1] Ser[1]
Bloomington Stock Center
11622
