# Supplementary material for: Machine Learning Analysis Identifies Drosophila Grunge/Atrophin as an Important Learning and Memory Gene Required for Memory Retention and Social Learning
Source: G3 (Bethesda). 2017 Sep 9;7(11):3705–18. doi: 10.1534/g3.117.300172 (PMC5677163; doi:10.1534/g3.117.300172)
Supplement: Supplementary file 12 [file 3705FileS3.pdf]

Table S1. Fly genotypes used in this study. Name, genotype, acquisition location, and stock identification number (if applicable) are shown.

File S1. Gene list of positive and negative standards used in PILGRM analysis. Genes in positive standard are indicated with a (1). Genes in negative standard indicated by a (-1).

File S2. Novel genes as predicted by PILGRM. Output gene list from PILGRM analysis.

Figure S1. Further evidence demonstrating the role of *Gug* in memory retention. Proportion of eggs laid on 6% ethanol oviposition cap following wasp exposure after three 24-hour time points is shown. Controls outcrossed to Canton S continue to oviposit on 6% ethanolafter wasp exposure. Outcrossed genotypes shown are OK107/+ (A), MB247/+ (B), *Gug*RNAi (C),and MBswitch/+ fed RU486. Error bars represent 95% confidence intervals (n = 10 biologicalreplicates) (*p < 0.05).

Figure S2. Collective evidence demonstrating the role of *Gug* in memory retention. Proportion of eggs laid on 6% ethanol oviposition cap following wasp exposure after three 24-hour time points is shown utilizing line graphs. These data are presented as bar graphs in Figure 3 and Figure S1. All control lines are shown in light blue, while all exposed treatments are shown in varying colors. *Gug* perturbation leads to an accelerated memory decay with the curves going down to unexposed levels.

Figure S3. Further evidence demonstrating the role of *Gug* in egg-retention memory. Percentage of eggs laid by exposed flies normalized to eggs laid by unexposed flies is shown. Wild-type flies exposed to wasps lay fewer eggs than unexposed flies for multiple days (A). *Gug*RNAi/+ (A) and His-RFP flies have wild-type memory retention (B). Error bars represent standard error (n = 24 biological replicates) (*p < 0.05).

Figure S4. Collective evidence demonstrating the role of *Gug* in egg retention memory. Percentage of eggs laid by exposed flies normalized to eggs laid by unexposed flies is shown utilizing line graphs. The data are also presented in bar graph form in Figure 4 and Figure S3. All control treatments are shown in light blue, while exposed treatments are shown in varying colors. *Gug* perturbation leads to an accelerated memory decay, with the curves going up to unexposed levels.

Figure S5. Further evidence demonstrating the role of *Gug* in teaching behavior. Percentage of eggs laid by exposed flies normalized to eggs laid by unexposed flies is shown. *Gug*RNAi/+ (outcrossed to Canton S) flies exposed to wasps can teach multiple student cohorts (RFP-Histone) across three days (A). Error bars represent standard error (n = 24 biological replicates) (*p < 0.05).

Figure S6. Collective evidence demonstrating the role of *Gug* in teaching behavior. Percentage of eggs laid by exposed flies normalized to eggs laid by unexposed flies is shown utilizing line graphs. The data are also presented in bar graph form in Figure 5 and Figure S4. All control treatments are shown in light blue, while exposed treatments are shown in varying colors.

A paired teacher-student is shown in the same color, with the teacher being lighter, and the student being darker (see legend). *Gug* perturbation leads to an accelerated memory decay and a loss of teaching efficiency, with both curves going up to unexposed levels.

Figure S7. Further evidence demonstrating the role of *Gug* in social learning. Percentage of eggs laid by exposed flies normalized to eggs laid by unexposed flies is shown. Wild-type flies (His-RFP) exposed to wasps can teach *Gug*RNAi/+ students (A). Error bars represent standard error (n = 24 biological replicates) (*p < 0.05).

Figure S8. Collective evidence demonstrating the role of *Gug* in social learning. Percentage of eggs laid by exposed flies normalized to eggs laid by unexposed flies is shown in line graph form. The data are also presented in bar graph form in Figure 6 and Figure S7. All control treatments are shown in light blue, while exposed treatments are shown in varying colors. A paired teacher-student is shown in the same color, with the teacher being lighter, and the student being darker having a filled in circle (see legend). *Gug* perturbation leads to impaired social learning.
